# Supplementary material for: Reproducibility of knee extensor and flexor contraction velocity in healthy men and women assessed using tensiomyography: A study protocol
Source: PLoS One. 2022 Jan 6;17(1):e0262156. doi: 10.1371/journal.pone.0262156 (PMC8735606; doi:10.1371/journal.pone.0262156)
Supplement: S1 Appendix — (PDF) [file pone.0262156.s001.pdf]

Deutsche Gesellschaft
für Ernährung e. V.

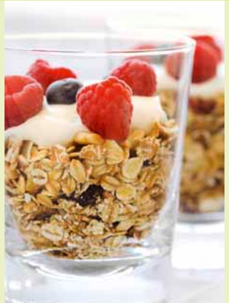
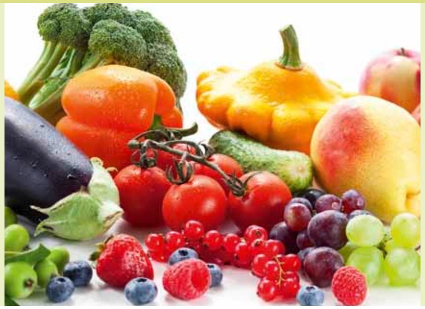
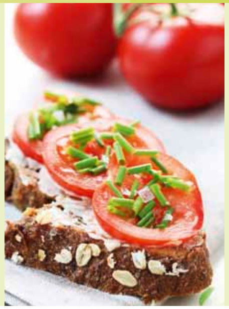
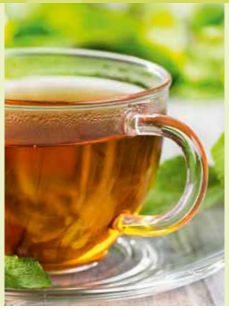
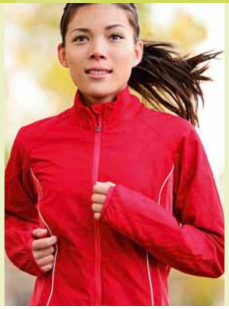

Mein Ernährungstagebuch

Name

Zeitraum

Ziel

Ihr Ernährungstagebuch – Hinweise zum Ausfüllen

Ein Ernährungstagebuch hilft Ihnen, Gewohnheiten zu erkennen. Nehmen Sie sich die Zeit und notieren Sie über eine Woche, was und wie viel Sie essen und trinken. Nach dieser Woche ziehen Sie Bilanz: Essen Sie ausreichend Gemüse und Obst? Trinken Sie genug? Verbringen Sie viel Zeit mit dem Essen vor dem Fernseher oder essen nebenbei? Was fällt Ihnen leicht, was können Sie verbessern?

Bei der Auswertung kann Ihnen eine Ernährungsfachkraft helfen. Adressen von qualifizierten Ernährungsfachkräften finden Sie unter „Service" auf der Website www.dge.de.

So führen Sie das Tagebuch:

- Notieren Sie auf dem Deckblatt Ihren Namen und den Zeitraum, in dem Sie das Ernährungstagebuch ausfüllen. Formulieren Sie ein Ziel für diesen Zeitraum.
- Füllen Sie täglich ein Blatt aus. Sollten Sie mehr Platz benötigen, nutzen Sie die Rückseite oder ein weiteres Blatt.
- Notieren Sie alles, was Sie essen und trinken – während einer Mahlzeit und auch nebenbei.
- Tragen Sie Ihre Angaben direkt nach dem Verzehr ein, dann vergessen Sie nichts.
- Beschreiben Sie alles so genau wie möglich. Geben Sie z. B. auch Fettgehalte von Milchprodukten an.
- Fehlen Ihnen Gewichtsangaben oder können Sie das Lebensmittel nicht abwiegen, dann schätzen Sie die Menge so gut wie möglich, z. B. 1 Teelöffel, 1 Scheibe, 1 Handtellergroß.
- Geben Sie bei den Getränken auch die Art an, z. B. Mineralwasser, Leitungswasser, schwarzer Tee, Fruchtsaft.
- Notieren Sie, wenn es Besonderheiten gab, z. B. Beschwerden nach dem Essen, Zeitdruck beim Essen, das Naschen im Auto. Auch Medikamente, die Sie nehmen, können Sie hier eintragen. Nutzen Sie dazu die Spalte „Anmerkungen/Situation".
- In der Spalte „Aktivitäten" können Sie Angaben zu Ihrer Bewegung machen. Hier zählt alles! Tragen Sie also nicht nur Ihre sportlichen Aktivitäten ein, sondern notieren Sie auch, wenn Sie die Treppe anstatt den Aufzug nutzen oder in der Mittagspause spazieren gehen.
- Unter „Notizen" können Sie alles aufschreiben, was Ihnen wichtig erscheint. Vielleicht mögen Sie ein Tagesmotto eintragen. An dieser Stelle ist auch Platz für spezielle Angaben, z. B. Arbeitstag oder Wochenende, Urlaubs- oder Krankheitstag, wann Sie aufgestanden und schlafen gegangen sind oder wie Sie sich an diesem Tag gefühlt haben.

Beispiel:

**UHRZEIT**<br>Wann?	**SPEISEN/MENGE**<br>Was?/Wie viel?	**GETRÄNKE/MENGE**<br>Was?/Wie viel?	**ANMERKUNGEN/SITUATION**<br>Wie habe ich mich gefühlt?	**AKTIVITÄTEN**<br>Wie viel habe ich mich bewegt?
7:00 Uhr	2 Scheiben Vollkornbrot, 2 Tl Butter, 1 Tl Konfitüre, 1 Scheibe Gouda 45 % F.i.Tr.	1 Tasse Kaffee (125 ml) 4 Tl Milch	etwas in Eile, zu Hause	mit dem Fahrrad zur Arbeit
10:30 Uhr	2 große Möhren (150 g), 1 Kaugummi	1 Glas Mineralwasser (250 ml)	Möhren haben gut geschmeckt, Büro	Treppe anstatt Aufzug genommen!
13:30 Uhr	1 Wiener Schnitzel (2 Handtellergroß), paniert und frittiert,<br>2 Schöpfkellen Kartoffelsalat.	1 Glas Mineralwasser (400 ml)	hektisch gegessen, wegen Besprechungstermin, Kantine	Treppe!
14:00 Uhr	3 Schokoplätzchen	2 Tassen Kaffee (125 ml), 2 Portionsdöschen Kaffeemilch leicht, 4 Tl Zucker, 1 kleine Flasche Mineralwasser (250 ml)	Besprechung, unangenehmes Bauchgefühl gehabt	
...				

Artikel-Nr.: 121515; 1. Auflage 2014
Bildnachweis: ©Fotolia.com: jillchen; sarsmis; Barbara Pheby; Nitr; Maridav

Mein Ernährungstagebuch

Name **Datum** 25.06.2021

Deutsche Gesellschaft für Ernährung e. V.

**UHRZEIT** Wann?	**SPEISEN/MENGE** Was?/Wie viel?	**GETRÄNKE/MENGE** Was?/Wie viel?	**ANMERKUNGEN/SITUATION** Wie habe ich mich gefühlt?	**AKTIVITÄTEN** Wie viel habe ich mich bewegt?

NOTIZEN

Mein Ernährungstagebuch

Name **Datum**

Deutsche Gesellschaft für Ernährung e. V.

**UHRZEIT** Wann?	**SPEISEN/MENGE** Was?/Wie viel?	**GETRÄNKE/MENGE** Was?/Wie viel?	**ANMERKUNGEN/SITUATION** Wie habe ich mich gefühlt?	**AKTIVITÄTEN** Wie viel habe ich mich bewegt?

NOTIZEN

Mein Ernährungstagebuch

Name **Datum**

Deutsche Gesellschaft für Ernährung e. V.

**UHRZEIT**<br>Wann?	**SPEISEN/MENGE**<br>Was?/Wie viel?	**GETRÄNKE/MENGE**<br>Was?/Wie viel?	**ANMERKUNGEN/SITUATION**<br>Wie habe ich mich gefühlt?	**AKTIVITÄTEN**<br>Wie viel habe ich mich bewegt?

NOTIZEN

Mein Ernährungstagebuch

Name **Datum**

Deutsche Gesellschaft für Ernährung e. V.

**UHRZEIT**<br>Wann?	**SPEISEN/MENGE**<br>Was?/Wie viel?	**GETRÄNKE/MENGE**<br>Was?/Wie viel?	**ANMERKUNGEN/SITUATION**<br>Wie habe ich mich gefühlt?	**AKTIVITÄTEN**<br>Wie viel habe ich mich bewegt?

NOTIZEN

Mein Ernährungstagebuch

Name **Datum**

Deutsche Gesellschaft
für Ernährung e. V.

**UHRZEIT**<br>Wann?	**SPEISEN/MENGE**<br>Was?/Wie viel?	**GETRÄNKE/MENGE**<br>Was?/Wie viel?	**ANMERKUNGEN/SITUATION**<br>Wie habe ich mich gefühlt?	**AKTIVITÄTEN**<br>Wie viel habe ich mich bewegt?

NOTIZEN

Mein Ernährungstagebuch

Name **Datum**

Deutsche Gesellschaft für Ernährung e. V.

**UHRZEIT** Wann?	**SPEISEN/MENGE** Was?/Wie viel?	**GETRÄNKE/MENGE** Was?/Wie viel?	**ANMERKUNGEN/SITUATION** Wie habe ich mich gefühlt?	**AKTIVITÄTEN** Wie viel habe ich mich bewegt?

NOTIZEN

Mein Ernährungstagebuch

Name **Datum**

Deutsche Gesellschaft für Ernährung e. V.

**UHRZEIT**<br>Wann?	**SPEISEN/MENGE**<br>Was?/Wie viel?	**GETRÄNKE/MENGE**<br>Was?/Wie viel?	**ANMERKUNGEN/SITUATION**<br>Wie habe ich mich gefühlt?	**AKTIVITÄTEN**<br>Wie viel habe ich mich bewegt?

NOTIZEN
