# Supplementary material for: Reproducibility of knee extensor and flexor contraction velocity in healthy men and women assessed using tensiomyography: A study protocol
Source: PLoS One. 2022 Jan 6;17(1):e0262156. doi: 10.1371/journal.pone.0262156 (PMC8735606; doi:10.1371/journal.pone.0262156)
Supplement: Attachment — Submitted filename: review PONE-D-21-24120.docx [file pone.0262156.s003.docx]

PONE-D-21-24120

Reproducibility of knee extensor and flexor contraction velocity in healthy men and women assessed using tensiomyography: A study protocol

General comment

Thank you for your thesis. Your research is very useful in establishing the reliability of TMG's methods. This study will be to investigate the within and between-day reliability of five different formulas to calculate Vc. The second aim will be to investigate the effect of different ISI during repeated stimulation on Vc, assessed by the example of M. biceps femoris and M. rectus femoris. However, there are some concerns that you should modify. Please refer following comments.

Line 107

Please explain the reason of selection in muscle (RF and BF) for your study.

Line 127

Please describe the location (setting) of your planned research.

Line 134

The description of IPAC-SF should be combined with the inclusion criteria of line129.

Line 141

When RF and BF are first mentioned, please use the formal names.

Line 142

Why will you perform your study with ten stimuli? Please add the reason the number of the stimuli in method.

Line 195

Please consider whether you will measure the thickness of the skin or subcutaneous tissue at the TMG measurement point. This may affect the measurement.
